# Diversity in Protein Profiles of Individual Calcium Oxalate Kidney Stones

**DOI:** 10.1371/journal.pone.0068624

**Published:** 2013-07-09

**Authors:** Nobuaki Okumura, Masao Tsujihata, Chikahiro Momohara, Iwao Yoshioka, Kouzou Suto, Norio Nonomura, Akihiko Okuyama, Toshifumi Takao

**Affiliations:** 1 Laboratory of Homeostatic Integration, Division of Integrated Protein Functions, Institute for Protein Research, Osaka University, Suita, Osaka, Japan; 2 Laboratory of Protein Profiling and Functional Proteomics, Research Center for Structural and Functional Proteomics, Institute for Protein Research, Osaka University, Suita, Osaka, Japan; 3 Department of Urology, Osaka University Graduate School of Medicine, Suita, Osaka, Japan; Federal University of São Paulo (UNIFESP), Escola Paulista de Medicina, Brazil

## Abstract

Calcium oxalate kidney stones contain low amounts of proteins, some of which have been implicated in progression or prevention of kidney stone formation. To gain insights into the pathophysiology of urolithiasis, we have characterized protein components of calcium oxalate kidney stones by proteomic approaches. Proteins extracted from kidney stones showed highly heterogeneous migration patterns in gel electrophoresis as reported. This was likely to be mainly due to proteolytic degradation and protein-protein crosslinking of Tamm-Horsfall protein and prothrombin. Protein profiles of calcium oxalate kidney stones were obtained by in-solution protease digestion followed by nanoLC-MALDI-tandem mass spectrometry, which resulted in identification of a total of 92 proteins in stones from 9 urolithiasis patients. Further analysis showed that protein species and their relative amounts were highly variable among individual stones. Although proteins such as prothrombin, osteopontin, calgranulin A and calgranulin B were found in most stones tested, some samples had high contents of prothrombin and osteopontin, while others had high contents of calgranulins. In addition, calgranulin-rich stones had various neutrophil-enriched proteins such as myeloperoxidase and lactotransferrin. These proteomic profiles of individual kidney stones suggest that multiple systems composed of different groups of proteins including leucocyte-derived ones are differently involved in pathogenesis of individual kidney stones depending on situations.

## Introduction

Urolithiasis is a common kidney disease with lifetime incidences of more than 10% of men and 5% of women in many countries with a few regional discrepancies. Although shock wave lithotripsy and endoscopy have now been available to remove kidney stones in a non-invasive way, the recurrence rate of urolithiasis is very high especially after shock wave treatment [1]. To reduce the occurrence and recurrence of kidney stone diseases, it is essential to know the pathophysiology of kidney stone formation, but it still remained unsolved how kidney stones are formed and how stone formation can be prevented.

Kidney stones of more than 60% of urolithiasis patients contain calcium oxalate (CaOx) as the major constituent. They are formed by complex processes including crystal nucleation, crystal growth, crystal aggregation, and crystal-cell interaction [1]. Formation of CaOx crystals could be initiated under supersaturation of CaOx, but healthy individuals do not develop kidney stones even though urinary CaOx is often supersaturated [2]. In addition, small urinary CaOx crystals are found not only in stone formers but also in those who have no urolithiasis history. These indicate that there are physiological systems that prevent CaOx stone formation in the kidney.

CaOx kidney stones generally contain a variety of proteins at low levels [3]. Proteins so far identified in CaOx kidney stones include Tamm-Horsfall protein (THP or uromodulin) [4], osteopontin [5], prothrombin [6], calgranulin A (S100A8) [7], calgranulin B (S100A9) [7], serum albumin [8], defensin [8], protein Z [9], and several leucocyte-specific proteins including cathepsin G, leucocyte elastase, azurocidin, eosinophil cationic protein, lysozyme, [10], and myeloperoxidase [11]. Recently, comprehensive analyses of kidney stones have been carried out using proteomic techniques and more protein species have been found in CaOx kidney stones [12]–[14].

It is to be noted that some of these proteins have inhibitory or stimulatory activity on stone formation, indicating that these may have key roles in pathophysiology of urolithiasis [15], [16]. Prothrombin fragment 1, which is the N-terminal region of prothrombin containing γ-carboxyglutamate (Gla) residues, is one of the most potent inhibitor of CaOx kidney stone formation [17], [18]. Calgranulin A and B [7], which are S100 family calcium binding proteins with EF-hand motifs, and osteopontin [19], [20], which is present on the apical surface of the urinary tract, also inhibit initiation and growth of CaOx crystals. On the other hand, THP has been reported to promote or inhibit kidney stone formation depending on conditions [21]. Recently, THP-null mice have high occurrence of calcium oxalate and calcium phosphate kidney stone formation, indicating that THP also inhibits stone formation in normal kidney [22], [23].

Although CaOx kidney stones contain a variety of proteins, conventional biochemical approaches are not satisfactory to analyze because of the low abundance of proteins and low solubility of CaOx. In addition, stone extract proteins commonly show highly diffused migration patterns in SDS-polyacrylamide gel electrophoresis (SDS-PAGE), making the analysis of stone proteins more difficult. In the last few years, proteomic approaches using nano-liquid chromatography (nanoLC) and tandem mass spectrometry (MS/MS) have been applied to study kidney stones [12]–[14]. In the present study, we have identified protein components in solubilized CaOx stones using nano-LC/MALDI-MS/MS, and analyzed the differences in protein components in individual CaOx stones. The results will provide information on the mechanisms of CaOx crystal formation and the biological systems that prevent the growth of CaOx urinary stones.

## Materials and Methods

### Ethics Statement

The research project presented in this paper was approved by the Institutional Review Board of the Graduate School of Medicine, Osaka University. Written informed consent from all subjects was obtained according to the procedure approved by the board.

### Preparation of Stone Extracts

Human kidney stones were obtained from 9 urolithiasis patients and the mineral components were determined by IR spectroscopy. Kidney stones that have CaOx monohydrate or dihydrate as the main component were used for the following study. The CaOx kidney stones weighing 10–20 mg each were washed three times with distilled water using a vortex mixer, and crushed to fine powder using an ultrasonic cell disruptor (Branson sonifier 250, Branson, Danbury, USA) in 50 volumes of solubilizing solution consisting of 10 mM Tris-HCl, pH 7.4, 1 mM dithiothreitol (DTT) and 1 mM phenylmethylsulfonyl fluoride. To a 3 ml aliquot of the suspension, 30 ml of 10 mM Tris-HCl, pH 8.0, containing 50 mM ethylenediaminetetraacetate (EDTA), 7 M urea, 2 M thiourea, and 10 mM DTT, were added and incubated for 3 hours at room temperature with constant shaking. The suspension was dialyzed against 2 litters of 10 mM Tris-HCl, pH 7.4 containing 1 mM DTT overnight at 4°C, then concentrated by ultrafiltration to about 50 µl (Amicon Ultra-15, Millipore, Billerica, USA). Protein concentration was determined by the method of Bradford [24] using bovine serum albumin as the standard.

### Sodium Dodecyl Sulfate-polyacrylamide Gel Electrophoresis (SDS-PAGE) and in-gel Trypsin Digestion

A stone extract containing 0.2 µg protein was separated by SDS-PAGE by the method of Laemmli [25], and stained with silver [26] or Sypro Ruby (Invitrogen) [27] as indicated in the figure legend. For in-gel trypsin digestion, the sample lane was cut into 14 pieces and the gel pieces were subjected to in-gel digestion with 1 pmol trypsin (Promega, Madison, USA) as described previously [28]. The resultant peptide mixture was applied on a 0.1 ml hydrophobic cartridge column (Intersep RP1, GL Science, Tokyo, Japan), washed with 0.1% trifluoroaceticacid (TFA) and eluted with 50% acetonitrile/0.1% TFA. The eluate was concentrated by a vacuum concentrator and dissolved in 25 µl of 5% acetonitrile/0.1% TFA.

### Enzyme Digestion of Stone Extracts in Solution

For shotgun analysis, each kidney stone extract (10 µg protein in 10 µl of 50 mM sodium chloride, 25 mM Tris-HCl, pH 7.4) was incubated with 0.5 unit of N-glycanase (Roche, Basel, Switzerland) overnight at 37°C. To this sample, 50 µl of denaturing solution consisting of 10 mM Tris-HCl, pH 7.4, 7 M urea, 2 M thiourea and 10 mM DTT was added and incubated for 30 min at room temperature. Then, cysteine residues were alkylated by adding 1 M acrylamide to the solution to give a final concentration of 50 mM, and incubated for 30 min at room temperature. The solution was dialyzed against 10 mM Tris-HCl containing 1 mM DTT overnight at 4°C, and concentrated by ultrafiltration using Microcon 30 (Millipore). The sample was incubated with 1 pmol trypsin (Promega) or chymotrypsin (Roche) overnight at 37 °C. The reaction mixture was desalted using a 0.1 ml hydrophobic cartridge column (Intersep RP1) as described above.

### Protein Identification by Nano-LC/MALDI-MS/MS and Statistical Analysis

The concentrated peptide mixture was separated on a C_18_ column (0.075×150 mm) using a nano-LC system (Ultimate, Dionex, Sunnyvale, USA). In detail, 10 µl of the sample was applied on the column, washed with 97% solvent A (0.1% TFA)/3% solvent B (acetonitrile/0.1% TFA), and eluted with an 80 min gradient of 3–80% solvent B at a flow rate of 300 nl/min. Each 1 min fraction was collected directly on a 192 well MALDI target plate using Probot Microfraction Collector (Dionex), and analyzed by a MALDI-MS/MS mass spectrometer (4700 Proteomics Analyzer, Applied Biosystems, Foster City, USA) with α-cyano-4-hydroxycinnamic acid as the matrix. From the resultant MS/MS spectra, a peak list was created by a software Peak to Mascot (ABI) with the following parameters; minimum S/N: 12, minimum area: 1000, and maximum peak/precursor: 100. Database search was carried out using MASCOT 1.0 search program (Matrix Science, London, UK) with following parameters; database: swissprot (release 54.5), taxonomy: human (containing 17742 sequences), peptide tolerance: 0.1 Da, peptide charge: 1+, MS/MS tolerance: 0.2 Da, fixed modifications: propionamide (C), Enzyme: none, variable modifications: oxidation (M), formylkynurenine (W) and deamidation (N), and no limitation of protein mass. A protein candidate was judged as a protein hit if the protein score was over 50 and at least one peptide was matched with an MS/MS ion score over 30. Principal component analysis was carried out using the function “prcomp” of the package “stat” of the software “R” (the R Foundation of the GNU Project).

### Immunoblotting

Kidney stone extracts containing 0.1 µg protein each were separated by SDS-PAGE using a 10% polyacrylamide gel with a cathode buffer consisting of 82 mM Tris, 100 mM Tricine and 0.1% SDS and an anode buffer consisting of 100 mM Tris-HCl, pH 8.8. For immunoblotting, proteins were transferred to a polyvinylidene fluoride membrane, and incubated in T-TBS (10 mM Tris-HCl, pH 7.4, 150 mM NaCl, and 0.1% Tween 20) containing 1% bovine serum albumin overnight at room temperature. The membrane was then incubated with a primary antibody diluted in T-TBS. The primary antibodies used were sheep anti-prothrombin fragment 1 (1∶50,000 dilution, Cedarlane, Ontario, Canada, CL20111AP), mouse anti-calgranulin A (1∶200 dilution, Santa Cruz Biotechnology, Santa Cruz, USA, sc-48352), goat anti-calgranulin B (1∶100 dilution, Santa Cruz Biotechnology, sc-8114), and rabbit anti-THP (1∶200, Santa Cruz Biotechnology, sc-20631) antibodies. After washing with T-TBS, the membrane was incubated with horseradish peroxidase-labeled anti-sheep IgG, anti-goat IgG, or anti-rabbit IgG. The membrane was washed with T-TBS, incubated with chemiluminescence reagents (Western lightning™, Perkin Elmer Life Sciences, Waltham, USA), and immunoreactive bands detected using an image analyzer with a cooled CCD detector (LAS-1000, Fujifilm, Tokyo, Japan). Images were saved in 16 bit Tiff format and the band intensities quantified using Image J software (National Institute of Health, Maryland, USA).

## Results

### Preparation of Kidney Stone Extracts and their Migration Patterns in SDS-PAGE

Calcium oxalate kidney stones were collected from nine urolithiasis patients. Infrared spectroscopic analysis showed that the major mineral component of sample 1 and 2 was calcium oxalate monohydrate, while that of sample 3–9 was calcium oxalate dihydrate (Table 1). Each kidney stone was crushed with an ultrasonic cell disruptor, and solubilized in a Tris-HCl buffer containing EDTA, urea, thiourea and DTT. To solubilize CaOx, EDTA was essential, while urea and thiourea were added to increase the solubility of low-solubility proteins. A few insoluble materials were visible after EDTA treatment, but they were disappeared after protease treatment (data not shown). Protein yields were ranging from 0.5 to 3 µg protein/mg kidney stone, except for Sample 4 that contained less than 0.1 µg protein/mg kidney stone (Table 1).

**Table 1:** Kidney stone samples analyzed in this study.

Sample number	Gender	Crystal form	Protein(µg/mg stone)
1	Female	COM	0.5
2	Male	COM	1.1
3	Male	COD	2.0
4	Male	COD	<0.1
5	Male	COD	3.0
6	Male	COD	3.0
7	Male	COD	1.8
8	Male	COD	2.2
9	Male	COD	2.6

**Figure 1:**
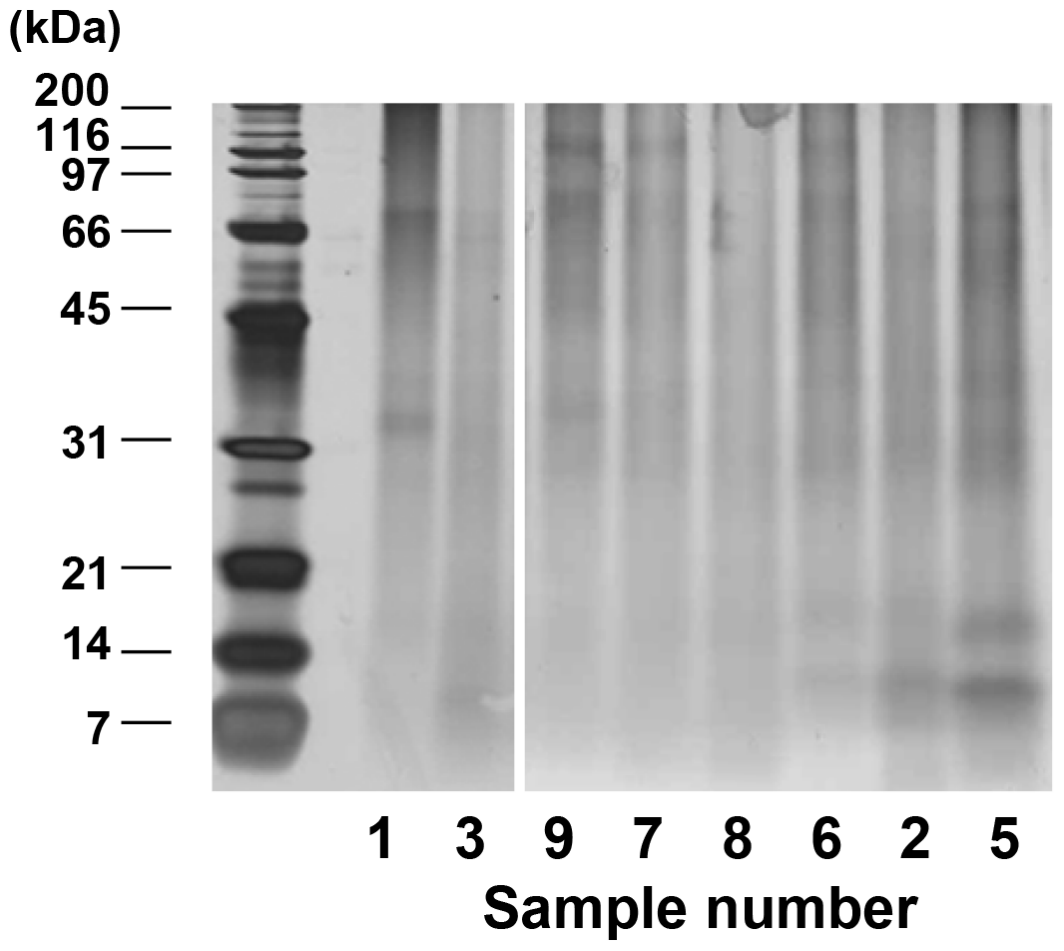
Analysis of proteins extracted from CaOx kidney stones by SDS-PAGE. CaOx kidney stones obtained from 8 individuals were solubilized, dialyzed, concentrated, and then the extracts containing 0.2 µg protein each were subjected to SDS-PAGE. Proteins were visualized by silver staining. Sample 4, the yield of which was <0.1 µg/mg stone, also showed similar diffused protein pattern (data not shown). Electrophoresis and staining were carried out three times, and all gave similar results.

Initial characterization of protein extracts from CaOx kidney stones was carried out using SDS-PAGE (Fig. 1). All extracts showed highly diffused staining patterns as reported by other groups [8], [9]. Only a few broad protein bands were detected at 30 kDa in sample 1 and 10–14 kDa in sample 6, 8, and 5, but the differences in protein patterns between samples were not clear.

**Figure 2:**
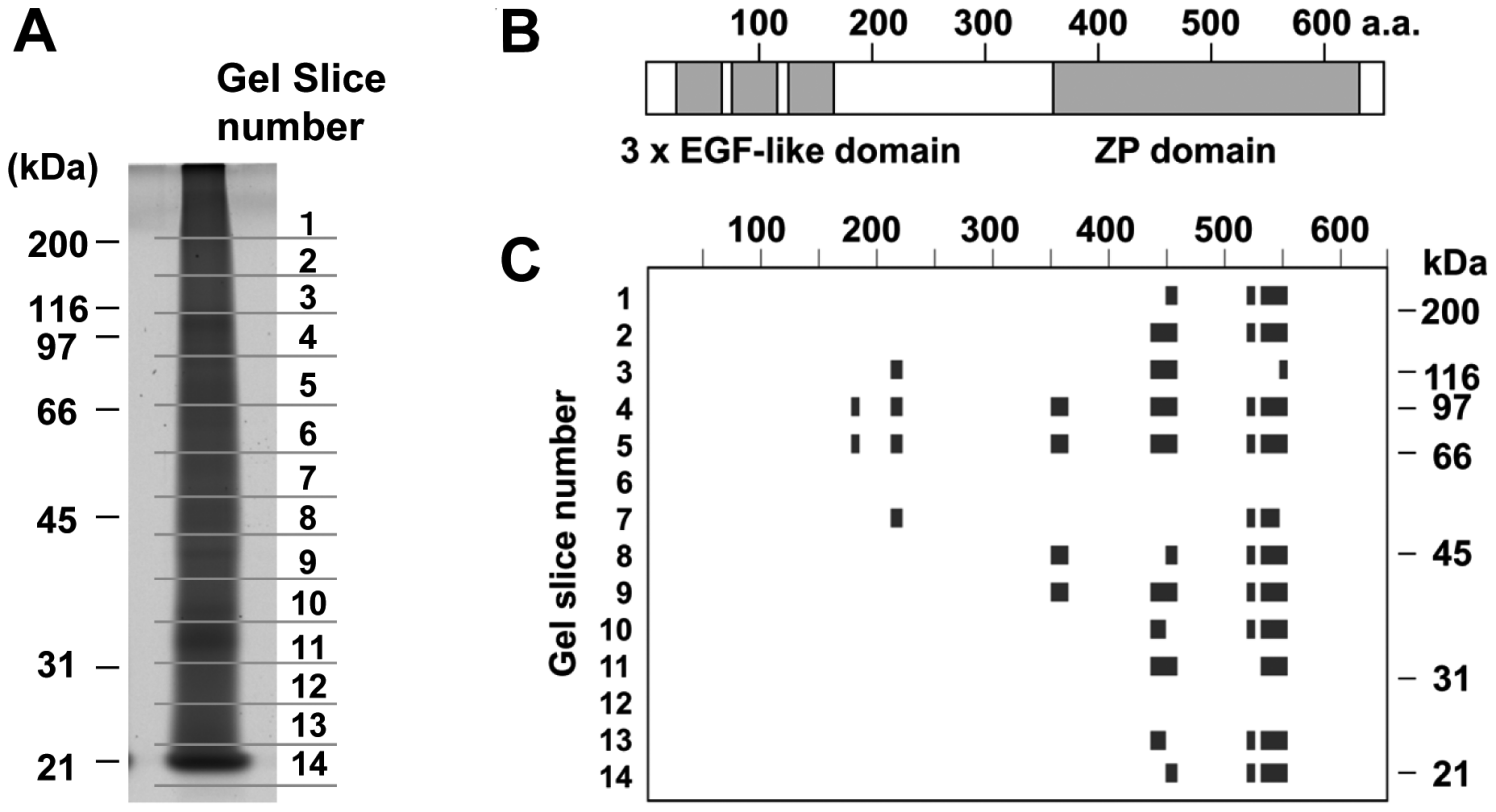
Characterization of gel-separated proteins from a CaOx kidney stone by nanoLC MALDI-MS/MS analysis. (A) One of the kidney stone extracts, Sample 3, was separated by SDS-PAGE, stained with Sypro Ruby, and cut into 14 pieces as indicated. Each gel piece was subjected to *in gel* trypsin digestion, and the resultant peptides were analyzed by nanoLC/MALDI MS/MS followed by database search on MASCOT. The results were presented as Table 2. (B) Domain structure of human THP (SwissProt : UROM_HUMAN). (C) From the results shown in Table 2, peptides matched to THP were mapped on the amino acid sequence of human THP.

To characterize the diffused protein patterns, one of the samples (Sample 3) was separated by SDS-PAGE, and divided into 14 parts according to electrophoretic mobility (Fig. 2A). We choose Sample 3 because it showed typical electrophoretic pattern (Fig. 1) and had moderate protein concentration (Table 1). The protein composition of each gel piece was analyzed by in-gel trypsin digestion followed by offline nanoLC/MALDI MS/MS analysis. The protein list of each gel piece was shown in Table 2, where Mascot scores together with the numbers of peptide hits were presented. From the 14 gel pieces, a total of 22 proteins were identified, and most of them were detected in specific regions of the gel. However, peptide fragments derived from prothrombin and THP were detected in almost entire area of the gel, indicating that at least these two proteins were highly heterogeneous in terms of electrophoretic mobility.

**Table 2:** Example of protein distribution in SDS-polyacrylamide gel*.

Protein name	SwissProt	Gel slice number													
	protein name	1	2	3	4	5	6	7	8	9	10	11	12	13	14
Prothrombin	THRB_HUMAN	76 (1)	76 (1)	75 (2)	151(3)	91 (2)	51 (2)	100(2)	115(2)	62 (2)	105(3)	192(5)	50 (2)		66 (2)
Tamm-Horsfall protein (Uromodulin)	UROM_HUMAN	51 (3)	59 (4)	46 (2)	189(9)	229(8)		42 (2)	134(5)	85 (3)	75 (3)	75 (3)		59 (2)	89 (4)
Vitamin K-dependent protein S	PROS_HUMAN		55 (1)		82 (4)	68 (3)		30 (1)							
Hemoglobin subunit beta	HBB_HUMAN	71 (2)													68 (2)
Keratin 1	K2C1_HUMAN				196(4)										
Keratin 9	K1C9_HUMAN				137(2)										
Keratin 10	K1C10_HUMAN				55 (1)										
Vitamin K-dependent protein Z	PROS_HUMAN				65 (3)										
Coagulation factor X	FA10_HUMAN				66 (2)						57 (2)				60 (2)
Antithrombin III	ANT3_HUMAN				38 (1)			73 (1)							
Protein SET	SET_HUMAN									90 (3)					
Splicing factor, arginine-serine rich 2	SFRS2_HUMAN										150(4)	33 (1)			
Splicing factor, arginine-serine rich 1	SFRS1_HUMAN										65 (1)				
Splicing factor, arginine-serine rich 7	SFRS7_HUMAN										71 (3)				
VItronectin	VTNC_HUMAN														67 (2)
High mobility group protein 1-like 10	HMG1X_HUMAN											30 (2)	42 (2)		
Heat shock protein beta-1	HSPB1_HUMAN												67 (3)		57 (2)
Splicing factor, arginine-serine rich 3	SFRS3_HUMAN														39 (1)
Mannan-binding lectin serine protease 2	MASP2_HUMAN	39 (2)				37 (1)								43 (1)	346(7)
Calgranulin A (S100-A8)	S10A8_HUMAN														175(4)
Calgranulin B (S100-A9)	S10A9_HUMAN														128(3)
Neutrophil defensin 3	DEF3_HUMAN														92 (3)
Bone marrow proteoglycan	PRG2_HUMAN														63 (3)
Complement C3	CO3_HUMAN														62 (4)
Actin, cytoplasmic 1	ACTB_HUMAN														71 (3)

* Extracts from Sample 3 was separated by SDS-PAGE, divided into 14 pieces (Fig. 2A), and analyzed by in-gel trypsin digestion followed by nanoLC-MALDI MS/MS analysis. In this table, Mascot scores together with the numbers of peptide hits (in the parenthesis) were presented.

To characterize the diffused migration patterns further, THP-derived fragments detected in each gel piece were mapped on its amino acid sequence (Fig. 2B, C). Fragments corresponding to the C-terminal zona pellucida-like (ZP) domain were detected in almost entire area of the gel, while the middle to N-terminal part was detected only in gel pieces 4–7. These results indicate that THP in CaOx kidney stones is present as heterogeneous forms by proteolytic degradation and crosslinking.

### Protein Profiling of CaOx Kidney Stone Extracts

**Figure 3:**
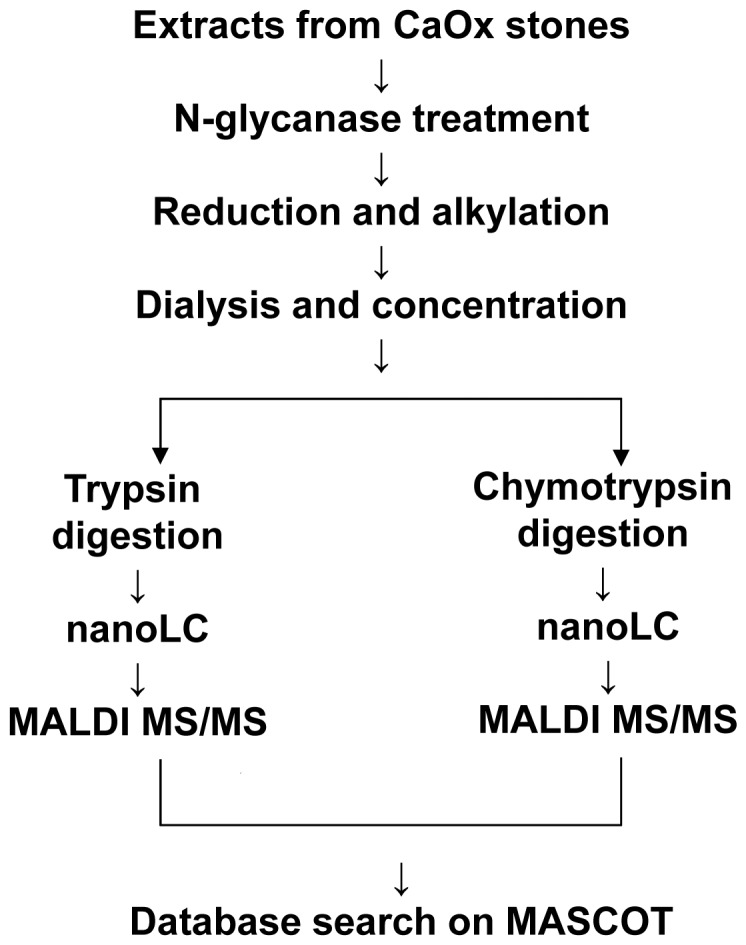
Procedure for protein profiling of CaOx renal stones. In order to analyze the protein compositions of individual CaOx stones, extracts from CaOx renal stones were subjected to protease digestion in solution and analyzed using nano-LC and MALDI-MS/MS as summarized in this scheme.

Since electrophoretic mobilities of certain kidney stone proteins were highly heterogeneous, protein profiles of individual CaOx kidney stones were obtained by in-solution protease digestion of protein extracts followed by nanoLC/MALDI-MS/MS analysis (Fig. 3). To further increase the number of peptide hits and peptide coverage, chymotrypsin digests in addition to trypsin digests were prepared and analyzed independently.

In this analysis, a total of 92 different proteins were identified in the 9 samples. The complete results of this analysis were shown in Table S1, where protein names, the number of peptide hits and protein coverages were presented, since the number of peptide hits and sequence coverages were expected to reflect the relative amount of the protein [29]. It is noteworthy that the protein list included at least 22 calcium-binding proteins that have a variety of calcium binding domains (Table S2) As shown in Fig. 3, trypsin- and chymotrypsin-digests were first analyzed by LC-MS/MS and peak lists obtained separately. When each peak list was subjected to database search, data from trypsin-digests resulted in more protein hits than those from chymotrypsin-digests (Table S3). However, combining the peak lists of trypsin- and chymotrypsin- digests resulted in increased protein hits (Table S3), increased protein score, and increased peptide match (data not shown). Therefore, combined peak lists were used in this analysis.

**Figure 4:**
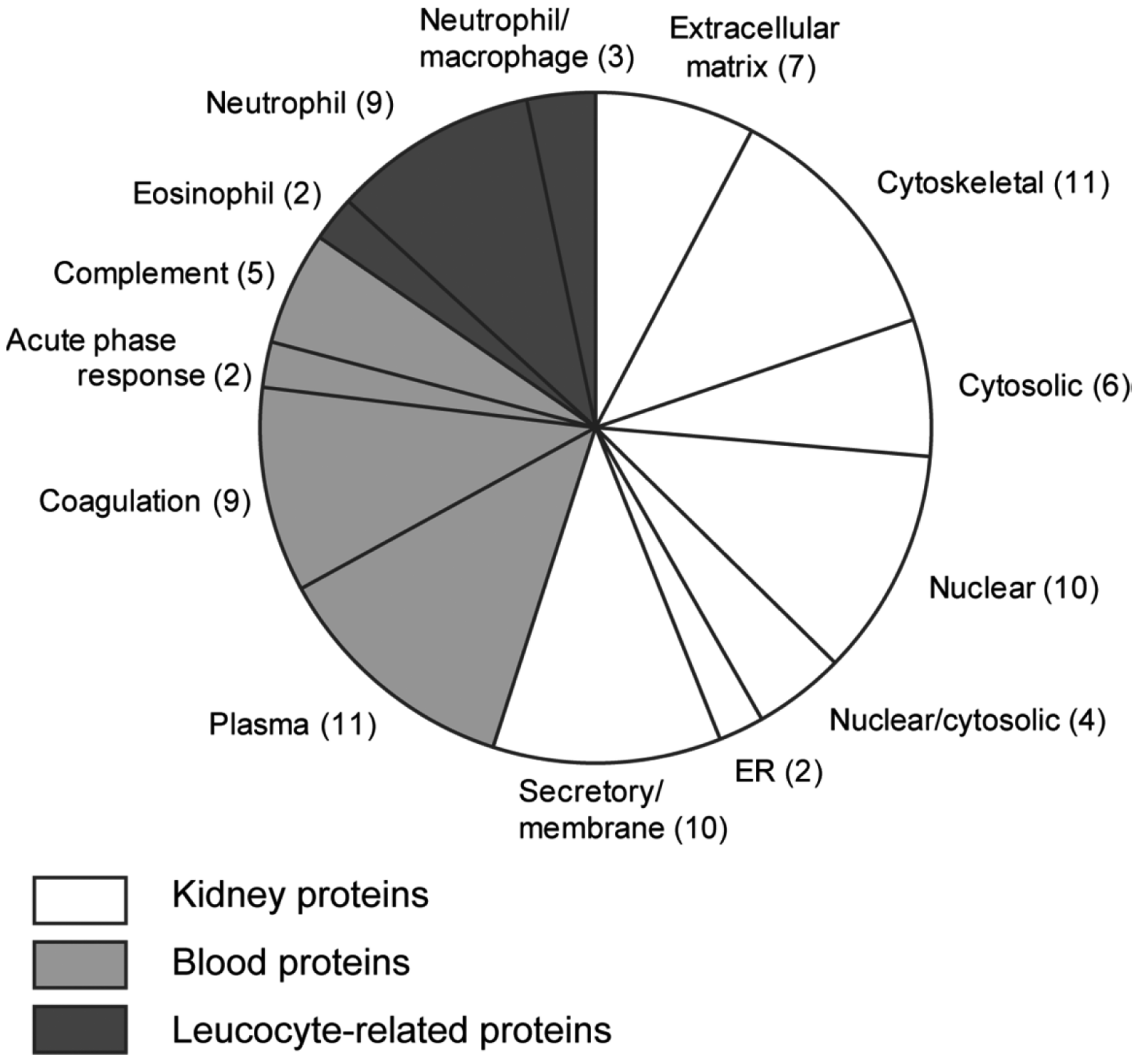
Classification of protein components detected in CaOx kidney stones. Extracts from 9 individual CaOx stone samples were examined separately by shotgun analysis as shown in Fig. 3, and the results in detail were presented in Table S1. As summarized in this figure, a total of 92 proteins were identified in this analysis. These proteins could be classified into three major groups according to their possible origins, i. e., kidney proteins, blood proteins and leucocyte-related proteins. Proteins in each group were further classified into subgroups.

Proteins found in CaOx kidney stones could be classified into three groups based on their possible origins (Fig. 4). Out of 92 proteins, 51 are those present in the intracellular regions or extracellular matrices of kidney cells. These include THP, which is expressed in the thick ascending limb of Henle (TALH), and osteopontin, which is in TALH and several other kidney regions such as the thin loop of Henle and the collecting duct. They also include vitronectin, fibronectin, and several proteoglycans, which are components of extracellular matrix or the basement membrane. All samples also contained cellular proteins that are abundant in the cytoskeleton (actin, myosin, tubulin etc.), cytosol (heat shock proteins, metabolic enzymes, etc.) and nucleus (nucleolin etc.) in most types of cells.

The second group is composed of proteins predominantly present in the blood. These include (1) major plasma proteins (e.g., serum albumin, immunoglobulin G) (2) coagulation system proteins (e.g., prothrombin, protein Z), (3) complement system proteins (e.g., mannan-binding lectin serine protease, complement C9), and (4) the acute phase response proteins (e.g., amyloid β A4 protein and serum amyloid P-component). Although these proteins are most likely derived from the blood, several proteins of this category such as prothrombin are also expressed in the kidney [30], thus we cannot exclude the possibility that some of these are originated from kidney cells.

The third group is composed of proteins highly expressed in leukocytes such as macrophages and neutrophils. These especially included a variety of proteins enriched in the azurophil granules of neutrophils [31] such as neutrophil defensin 3, α-HS-glycoprotein (fetuin A), lactotransferrin, azurocidin, myeloperoxidase and cathepsin G. Two eosinophil-specific proteins were also found in several samples. Calgranulin A and calgranulin B were present in most samples, and were especially abundant in Sample 2, 5, 6, and 8. These calgranulins are known to be expressed in monocytes, at high levels and thus were classified into the third group in this figure. However, some other types of kidney cells such as epithelial cells could also produce calgranulins in the kidney [7].

### Differences in protein compositions of Individual CaOx Kidney Stones

**Figure 5:**
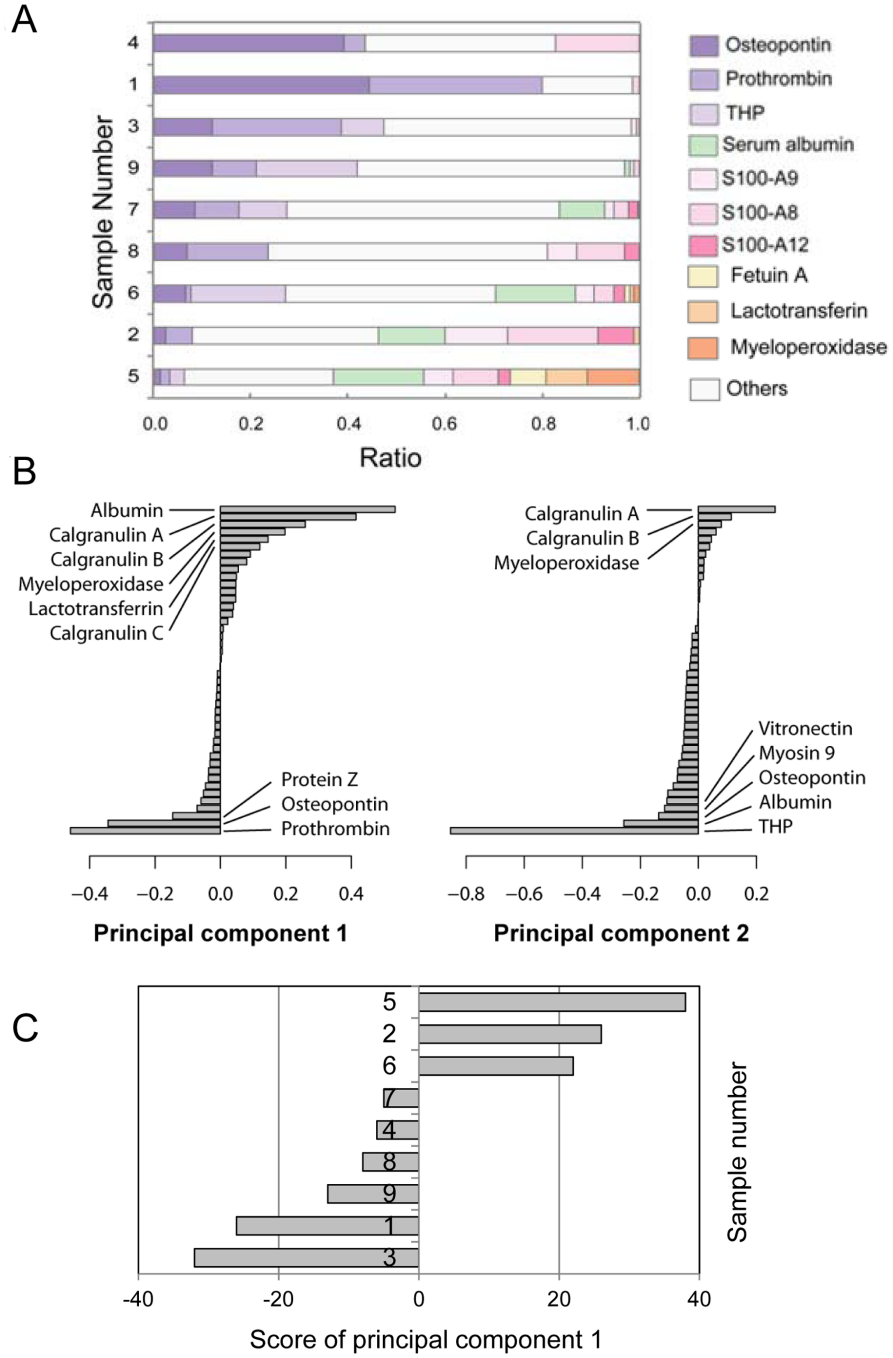
Comparison of relative peptide numbers detected of individual CaOx kidney stones. (A) After nano-LC/MALDI-MS/MS analysis, the numbers of peptide hits were presented as a bar graph with the total number of peptide hits in each gel sample as 100%. (B) The numbers of peptide hits were subjected to principal component analysis, and the first (left) and the second (right) eigenvectors were shown in a decreasing order. (C) The score of principal component 1 of each sample was presented as a bar graph.

This analysis also indicated that protein compositions of individual kidney stones are different each other (Table S1). To clarify the differences in protein compositions, percent ratios of the number of peptide hits of major kidney stone components were calculated (Fig. 5A). In sample 1, nearly 80% of total peptide hits were those derived from osteopontin and prothrombin. On the other hand, in sample 4, 3, and 9, peptides from these two proteins were 30–50%, and were less than 10% in sample 6, 2, and 5. In turn, calgranulins, serum albumin, and several neutrophil-specific proteins were predominant in sample 6, 2, and 5.

To further analyze the differences in protein components of individual kidney stones, the numbers of peptide hits were subjected to principal component analysis (Fig. 5B, C). In Component 1, proteins including serum albumin, calgranulin A, calgranulin B, myeloperoxidase, and lactotransferrin gave positive scores, while osteopontin, prothrombin, and vitamin K-dependent protein Z gave negative scores. On the other hand, Component 2 is largely dependent on the content of THP. With respect to component 1, sample 2, 5, and 6 showed positive scores, while sample 1, 3, 4, 7, 8 and 9 showed opposite scores (Fig. 5C). These results indicate that individual kidney stones have several different groups of proteins, and that the ratio of each group varies from sample to sample. In addition, it was suggested that osteopontin, prothrombin, and protein Z compose one group, while serum albumin, calgranulin A, calgranulin B, myeloperoxidase and lactotransferrin compose another group.

**Figure 6:**
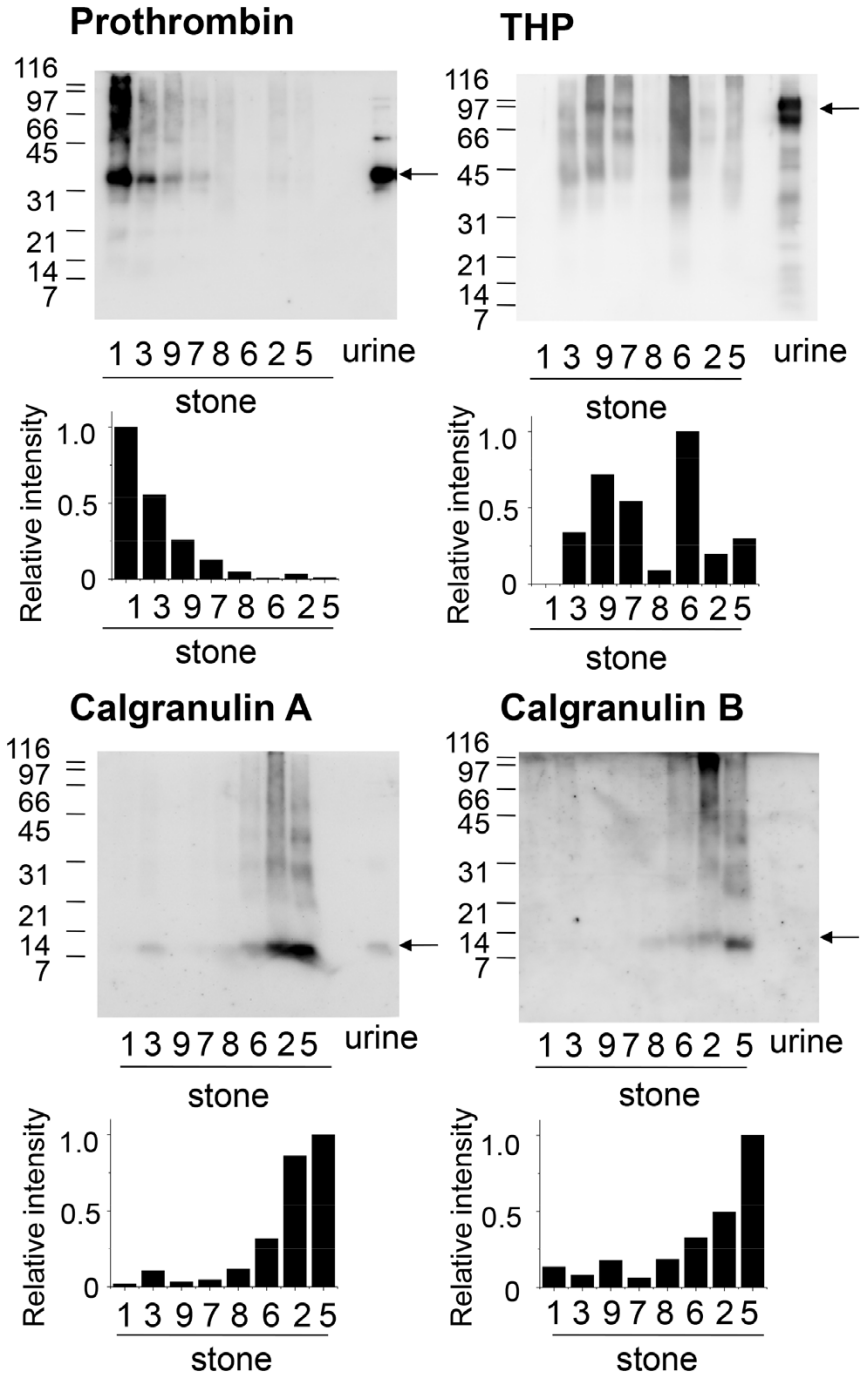
Western blot analysis of major proteins in CaOx kidney stones. Extracts of CaOx kidney stones containing 0.1 µg proteins were separated by SDS-PAGE and blotted on a PVDF membrane. To confirm the reactivity of antibodies and indicate the size of the proteins in urine, a concentrated urine sample from a normal subject (1 µg protein) was applied on the last lane of each gel. The membrane was then probed with anti-prothrombin fragment 1, anti-calgranulin A, anti-calgranulin B or anti-THP antibodies. Arrows indicate the positions of prothrombin fragment 1 (upper left), full length THP (upper right), full length calgranulin A (lower left), and full length calgranulin B (lower right). Western blotting was repeated two times and gave similar results. When Sample 1, 3, 9 and 7 (Group 1) was compared with Sample 8, 6, 2 and 5 (Group 2), p-value of Mann-Whitney U-test was 0.028 for prothrombin, calgranulin A and calgranulin B, while that for THP was 0.8857.

To further confirm the differences in protein profiles of individual kidney stones, prothrombin, calgranulin A, calgranulin B, and THP were examined by western blotting (Fig. 6). In order to confirm the specificity of the antibodies used, a concentrated urine sample from a normal subject was applied on the last lane of each gel, since these proteins have been known to be present in urine [32]–[34]. This also allows us to compare the molecular forms of the antigens in kidney stones with those in urine.

Prothrombin, the precursor of thrombin, is composed of fragment 1, fragment 2 and the thrombin protease domain. Using anti-prothrombin fragment 1 antibody, protein bands corresponding to fragment 1, which migrated just above 31 kDa, were detected in several kidney stone extracts as well as in urine [32]. The amount of prothrombin was highest in sample 1, followed by sample 3, while it was difficult to detect in sample 8, 6, 2 and 5. This figure also shows that kidney stone extracts, but not urine, had low mobility forms of prothrombin with apparent molecular weights of 45–200 kDa, indicating that there are cross-linked forms in kidney stones.

Calgranulin A and calgranulin B are small proteins with theoretical molecular weights of 11 k and 13 k, respectively. The western blot analysis confirmed that calgranulin A is present in normal urine and migrated at the position near its theoretical mass (Fig. 6). On the other hand, calgranulin B was not detected in normal urine in this analysis. It has been reported that urinary calgranulin B contents in men are much lower than those in women [33]. Since the normal urine we used was from a male, the absence of calgranulin B band might be due to this gender difference. In kidney stone extracts, calgranulin A and calgranulin B appeared as 8 and 12 kDa bands, respectively, with weak smear signals in the high molecular weight region. In contrast to prothrombin, their immunoreactive bands were highest in sample 5, followed by sample 2, 6, and 8. This reciprocal relationship between the protein levels of prothrombin and calgranulins is consistent with the results shown in Fig. 5.

A GPI-anchored membrane glycoprotein, THP, is the major urinary component with an apparent molecular weight of 60–90 kDa as shown in Fig. 2. In the kidney stone extracts, however, it migrated as a smear band of >30 kDa, indicated that it is degraded and cross-linked in kidney stones. This is consistent with the result of Fig. 2. The intensity of THP-immunoreactivity was highest in sample 6, followed by 9, 3, 5 and 7, and was not related to the amount of prothrombin and calgranulins. This is also consistent with the results shown in Fig. 5. When Sample 1, 3, 9 and 7 (Group 1) was compared with Sample 8, 6, 2 and 5 (Group 2) by Mann-Whitney U-test, prothrombin was significantly higher in Group 1 (p<0.05), while calgranulin A and calgranulin B was higher in Group 2 (p<0.05). In contrast, THP was not statistically different between the two groups as the p-value was 0.88.

## Discussion

In the present study, protein extracts from CaOx kidney stones have been characterized by nanoLC-MALDI analysis. First, we carried out initial characterization of kidney stone extracts using SDS-PAGE, and confirmed that proteins in kidney stones were highly heterogeneous in electrophoretic mobility (Fig. 1, 2). The diffused pattern of proteins seemed partly due to heterogeneity of major stone components, THP and prothrombin (Fig. 2B). In the case of THP, the C-terminal ZP domain was detected in the higher and lower region than the original migration position as detected by nanoLC-MALDI MS/MS spectrometry. These indicate that some proteins in CaOx kidney stones such as THP are influenced by proteolytic degradation and protein-protein crosslinking.

Since proteins in CaOx kidney stones were poorly separated by SDS-PAGE, protein profiles of individual kidney stones were investigated by shotgun analysis, and a total of 92 proteins have been identified in CaOx kidney stones from 9 patients. The protein list contained typical components of kidney stones such as prothrombin, THP, calgranulin A, calgranulin B, and myeloperoxidase [13]. In addition, it was noticed that protein compositions of individual samples are different each other as discussed below. Papers so far published sometimes show a few discrepancies, e.g., myeloperoxidase was detected in several studies, while it was not detected in others. The present results indicate that these discrepancies are likely to be due to the diversity of protein compositions of individual samples.

The proteins identified in the samples were classified into three groups. One of the groups contains those that are likely to be derived from kidney cells such as kidney tubular epithelial cells and interstitial cells. Osteopontin and THP are major proteins expressed on the apical surface of the renal tubules, while vitronectin, fibronectin, and proteoglycans are components of extracellular matrix. Fibronectin is induced by CaOx [34], and prevents aggregation of CaOx kidney stones [34], cell injury induced by CaOx [35] and endocytosis [36]. These results are consistent with the idea that certain cell surface molecules possibly function to prevent crystal growth and cellular damage.

The samples also had blood proteins including serum albumin, immunoglobulin gamma, and a variety of blood coagulation proteins such as prothrombin, protein Z, protein C, protein S, fibrinogens, plasminogen, and coagulation factor X. Although they are most likely derived from the blood, some of these proteins such as prothrombin are also expressed in the kidney [30]. It was reported that prothrombin, protein Z, protein S and osteopontin were present in CaOx stones, but not in uric acid stones, indicating that these have essential roles in CaOx stone formation [14]. Consistent with this, prothrombin and osteopontin were detected in all CaOx stones tested in the present study.

All CaOx kidney stones tested also contained variable amounts of calgranulin A and calgranulin B (Fig. 5, Table S1). These proteins are highly expressed in macrophages and neutrophils, and secreted from the cells. In addition, several calgranulin A-rich stones also contained neutrophil defensin 3, calgranulin C, fetuin A, lactotransferrin, azurocidin and myeloperoxidase, which are expressed principally in neutrophils [37]. The presence of these leucocyte-derived proteins in CaOx kidney stones is consistent with several other reports [11], [13]. These indicate that leucocytes are present near CaOx kidney stones in certain situations.

The differences in protein profiles of individual stones were further analyzed by several ways. A principal component analysis showed that principal component 1 was an axis created mainly by prothrombin, osteopontin and protein Z versus myeloperoxidase, calgranulin A and calgranulin B (Fig. 5B, C). Actually, prothrombin showed reciprocal relationship to calgranulin A and B as confirmed by western blotting (Fig. 6). These differences may reflect the presence or absence of calgranulin- and myeloperoxidase-producing cells near the stones. Macrophages or neutrophils are not typically observed near the site of stone formation, but a few lymphocyte infiltration has been reported by ethylene glycol-loaded rats [30]. In addition, early CaOx crystals produced by ethylene glycol loading is surrounded by epithelial cells and interacted with lymphocytes, indicating that there are active clearance mechanisms that remove CaOx deposits by these cells [38]. Therefore, the present study suggests that several different clearance systems including lymphocytes may be acting on CaOx stones depending on situations in human urolithiasis patient.

## Supporting Information

Table S1 **Protein profiles of CaOx kidney stones obtained from 9 patients.** * The numbers of peptide hits together with percent sequence coverage (in the parentheses).
(XLSX)

Table S2 **Metal-binding proteins detected in CaOx kidney stones.**
(XLSX)

Table S3 **Comparison of effectiveness of trypsin- and chymotrypsin-digestion for shotgun protein identification.** MS/MS peak lists obtained from trypsin- and chymotrypsin-digests were first separately subjected to database search analysis using MASCOT. After that, the two peak lists were combined and searched by the same way. The number of protein hits automatically detected by MASCOT (p<0.05) were presented in the table.
(XLSX)
